# Dendritic cells are susceptible to infection with wild-type adenovirus, inducing a differentiation arrest in precursor cells and inducing a strong T-cell stimulation

**DOI:** 10.1099/vir.0.013920-0

**Published:** 2010-05

**Authors:** Tobias Keßler, Klaus Hamprecht, Tobias Feuchtinger, Gerhard Jahn

**Affiliations:** 1 Institut für Medizinische Virologie und Epidemiologie der Viruserkrankungen, D-72076 Tübingen, Germany; 2 Universitätsklinik für Kinder- und Jugendmedizin, Eberhard Karls Universität Tübingen, Tübingen, Germany

## Abstract

Adenovirus infection after stem cell transplantation is a significant cause of morbidity and mortality, especially in children. A robust T-cell response induced by dendritic cells (DC) is crucial for clearing the virus, suggesting their pivotal role for the response to human adenoviruses (HAdV). Despite the widespread use of adenoviral vectors, the properties and kinetics of HAdV infection of DC have not been addressed yet. We show that a recent clinical HAdV, subgenus C/serotype 2 (strain BB2000-61), infects cells of the myeloid lineage. Infected DC produce early and late viral antigens and show an altered expression of surface markers. Infection of monocytes renders them refractory to differentiation into DC. Additionally, HAdV-infected DC are strong stimulators of CD8^+^ T cells. In summary, HAdV seems to manipulate the immune response by infection of DC and possibly uses the infection of monocytes as a means to escape recognition by T cells.

Human adenovirus (HAdV) frequently causes subclinical infections and is additionally associated with acute respiratory tract outbreak, epidemic kerato-conjunctivitis and acute gastroenteritis. Life threatening systemic infections occur in immunosuppressed patients after allogeneic stem cell transplantation (SCT) as well as solid organ transplantation. Incidence of HAdV infection is highest in children after allogeneic SCT. Risk factors are any cause of severe T-cell deficiency post SCT, like T-cell depletion of the graft, alternative donor SCT, delayed T-cell reconstitution and graft-versus-host-disease (Feuchtinger *et al.*, 2007; Howard *et al.*, 1999; La Rosa *et al.*, 2001; van Tol *et al.*, 2005). In contrast to the widespread use of adenovirus (AdV) for gene therapy or vaccination trials (Samulski *et al.*, 1987), data on infection of immunologically relevant cell types by clinical adenoviruses are sparse at best. It is yet unknown how the interplay between the innate and adaptive immune response is affected by HAdV, or how antigen presenting cells are modulated by the infection. Dendritic cells (DC) play a crucial role at the crossroad between innate and adaptive immune response as they can prime and activate T cells and modulate the type of T-cell response. This makes them an attractive target for viruses as many viruses are known to impair DC maturation and function. Monocyte-derived DC (mdDC) have evolved as an ideal cell type in order to study the effects of viral infections, for example human cytomegalovirus (HCMV) (Grigoleit *et al.*, 2002; Jahn *et al.*, 1999; Moutaftsi *et al.*, 2004; Raftery *et al.*, 2001, 2009; Riegler *et al.*, 2000). It was thought until very recently that there were no data on the susceptibility of DC for adenoviruses (Adams *et al.*, 2009). Langerhans cells and dermal DC express low levels of the coxsackievirus and adenovirus receptor (CAR), while mdDC lack this main receptor for the clinically most relevant subgroup of HAdV, Ad5. HAdV overcomes this limitation by using integrins as alternative entry receptors. Additionally, it has now been demonstrated that lactoferrin in association with DC-SIGN facilitates entry of recombinant HAdV5 into mdDC by mechanisms independent of CAR (Adams *et al.*, 2009).

Up to now there has not been any data about the interactions of clinical HAdV strains with DC and their capability to stimulate T cells. We investigated the susceptibility of DC and their precursor cells for wild-type adenovirus infection and analysed their T-cell stimulatory capacity. We infected monocytes, immature and mature mdDC with the clinical HAdV strain BB2000-61 (subgenus C/serotype 2). We isolated BB2000-61 from the blood of an SCT recipient (unpublished data). Subsequently, we analysed the expression of distinct surface markers in response to exposure to HAdV and finally tested the T-cell stimulatory capacity of HAdV-infected DC. It could be demonstrated that both monocytes and mdDC are susceptible to an infection with a clinical HAdV strain. The infection of immature mdDC resulted in an increased expression of major histocompatibility complexes (MHC) class I and class II, while expression of costimulatory molecules was heterogeneous. When mature mdDC were infected with BB2000-61, expression of MHC and costimulatory molecules remained stable or decreased. The infection of monocytes resulted in a block of differentiation towards immature DC. Finally, BB2000-61-infected DC stimulated CD8^+^ T cells.

Up to now there has not been any publication as to whether wild-type HAdV can infect DC. In order to address this question, we inoculated immature mdDC with BB2000-61. We analysed the replication of BB2000-61 and an HAdV reference strain of subgenus C/serotype 5 (provided by A. Heim, Medizinische Hochschule Hannover, Germany). DC were monocyte derived (Grigoleit *et al.*, 2002). In brief, peripheral blood mononuclear cells (PBMC) were separated from buffy coats by density-gradient centrifugation followed by CD14-based magnetic positive-selection of monocytes. Monocytes were used for experiments or incubated in six-well plates in RPMI with 10 % fetal calf serum, 100 μg gentamicin ml^−1^, 1000 U IL-4 ml^−1^ and 100 ng GM-CSF ml^−1^. After 7 days, immature DC were rinsed off the plates and controlled morphologically as well as for expression of CD1a, CD83, MHC I, MHC II, CD80, CD86 and CD40. For infection experiments, cells were incubated with different m.o.i. amounts of strain BB2000-61 and further cultivated. Thereafter, the cells were spun onto glass slides and stained for viral early (E) and late (L) antigens. Monoclonal antibodies (mAbs) were used directed against the E protein E1A (ab33183; Abcam; IgG2a; Cambridge) and the L hexon protein (C5000; Virion or Chemicon). Cytospins were fixed in acetone for 10 min at room temperature and incubated with the HAdV mAbs for 90 min at 37 °C, followed by incubation with Cy3-conjugated F(ab)′2 fragments of goat anti-mouse IgG antibodies (Jackson ImmunoResearch). Finally, cells were counterstained with 4,6-diamidino-2-phenylindole (DAPI).

Primary monocytes were susceptible to infection with the clinical HAdV strain as demonstrated by the expression of the E and L genes (Fig. 1). Additionally, immature mdDC could be infected with BB2000-61. When different m.o.i. amounts were compared for the resulting efficiency of infection, the percentage of infected cells ranged between 29 and 39 % at an m.o.i. between 1 and 50 with a peak efficiency of 39 % at an m.o.i. of 10. The range was the same for monocytes and mature mdDC. Intriguingly, higher m.o.i. amounts did not result in higher efficiencies of infection, but more cell debris was observed. The reference strain was also able to infect mdDC and monocytes. Further experiments were done with BB2000-61. This demonstrated for the first time that DC are a target for a clinical HAdV strain and both immature DC and their progenitors can be infected. Additionally, the infection did not abort and both the E and L proteins were expressed.

In order to assess if mature mdDC are also susceptible to infection with HAdV, we added 1 μg LPS ml^−1^ or 10 μg poly (I : C) (Sigma Aldrich) ml^−1^ after 7 days and cultivated them for a further 24 h. This induced DC maturation was demonstrated by upregulation of MHC class I, class II, CD40, CD80, CD86 and CD83 expression (data no shown). Mature DC were inoculated with BB2000-61 as described for immature DC and cultivated for a further 5 days. Mature mdDC had approximately the same susceptibility as immature mdDC. This shows that the myeloid lineage from monocytes to mature DC can be a target for HAdV, and replication at least reaches the late phase. The reference strain showed similar properties in infectivity, but was not further included in flow cytometry experiments/T-cell stimulation.

Many viruses are known to target DC and impair their function in order to subvert adaptive immunity. For example, HCMV infection impairs formation and intracellular transport of MHC molecules, resulting in a decreased surface expression of the respective molecules (Beck *et al.*, 2003; Grigoleit *et al.*, 2002; Kessler *et al.*, 2008; Raftery *et al.*, 2001; Tomazin *et al.*, 1999). In order to assess how a clinical HAdV strain acts in this regard, we examined the surface expression of immunologically relevant cell markers in mdDC after infection. To this end immature mdDC were infected with BB2000-61 and incubated for a further 5 days. Then the expression of various surface markers was analysed. Cytometry was performed using fluorescein isothiocyanate- or phycoerythrin-conjugated mAbs against surface markers: CD40 (Immunotech) and CD80, CD83, CD86, human leukocyte antigen (HLA)-DR, CD14, HLA-A, -B and C (W6/32), CD1a and IgG1 isotype control (Pharmingen). Infection of immature mdDC with HAdV results in a rather heterogeneous immunophenotype as some markers were upregulated, while others remained stable or were downregulated. In detail, expression of MHC class I and class II as CD86 increased, while CD80 was unaffected. CD83 was slightly downregulated, while CD1a was strongly reduced (Fig. 2a).

Manipulating the expression of functional surface markers in infected DC is a known mechanism of viral immune evasion (Grigoleit *et al.*, 2002; Schneider *et al.*, 2008). While HCMV as a prominent example causes a somewhat uniform downregulation of expression of both MHC and costimulatory molecules, HAdV induces a more heterogeneous picture. Both MHC class I and class II are upregulated as well as CD86 and CD40. All these molecules are important for a successful interplay between DC and T cells. On the other hand, infected mdDC show no upregulation of CD83, a marker for mdDC maturation.

As mature mdDC were also susceptible to HAdV, we analysed the immunophenotype. By impairing the surface expression of MHC and costimulatory molecules, HAdV could possibly interfere with T-cell priming and activation even after the initial activation of the DC. Therefore, we induced maturation of immature mdDC by the addition of 1 μg LPS ml^−1^ or 10 μg poly (I : C) (Sigma-Aldrich) ml^−1^. Cells were further cultivated and infected with BB2000-61 as described above. Stimulation with LPS and poly (I : C) resulted in an increased expression of MHC class II and a more pronounced expression of CD80 and CD86. Comparing the stimulatory performance, poly (I : C) was superior to LPS. Nevertheless, when HAdV-infected samples were compared with mock, HAdV infection resulted in a decreased surface expression of CD80 and CD86. Also, poly (I : C)-stimulated DC expression of MHC class II was decreased, while it remained stable in LPS-matured cells. Intriguingly, CD1a was expressed in higher amounts on HAdV-infected LPS-matured DC than in poly (I : C) stimulated ones.

LPS and poly (I : C) induce DC maturation by binding to TLR4 and TLR3, respectively. While TLR4 is located on the cell surface and common on many DC subsets, TLR3 resides in endosomes and is a unique feature of DC of the myeloid lineage. The different pattern recognition receptors, their localization and their signalling pathways possibly are reflected in the variable response to LPS and poly (I : C). Overall, maturation induced by poly (I : C) seems to be more thorough as reflected by a stronger upregulation of surface marker expression (Fig. 2c) and additionally is permanent. After infection with HAdV, the immunophenotype of mdDC matured with both LPS and poly (I : C) is skewed to the level or below of immature mdDC. The upregulation of MHC II and primarily CD80 and CD86 that accompanies DC maturation is reverted by HAdV. The infection and subsequent manipulation of mature DC could be one module of a multi-component immune evasion strategy by HAdV. As immature DC respond to HAdV infection with an upregulation of certain activating molecules, formerly uninfected mature DC could be impaired in their function. This would be especially relevant for DC cross-presenting antigens without direct contact to an antigen.

One important subtype of DC results from the differentiation of monocytes into immature DC under the influence of cytokines. As we demonstrated, monocytes are susceptible to infection with a clinical HAdV strain. Infection of the direct progenitor of the cell type that links innate and adaptive immunity could be beneficial to a virus, since it could modulate its differentiation process and function. To test this assumption, we infected monocytes and added the cytokines necessary for differentiation into immature mdDC. After 7 days in culture (i.c.), we compared the cells to both the freshly isolated monocytes and non-infected cells with the same treatment regarding their morphology and surface marker expression (Fig. 2b). Viewed by microscope, monocytes appear as round bright cells, while DC are less bright and feature their characteristic shape. The majority of the HAdV-infected monocytes remained as round and bright as freshly isolated monocytes even after 7 days i.c., while a small fraction showed the typical shape and colour of macrophages. When the freshly isolated mock-infected monocytes were compared with another fraction of the same monocytes that was differentiated into mdDC, the latter showed all signs of differentiation: CD1a, MHC class II, CD80, CD86 and CD40 expression was upregulated, while CD14 expression was almost diminished. In contrast, HAdV-infected cells showed no signs of differentiation into DC. CD1a, MHC class II, CD80, CD86 and CD40 expression were downregulated, with the exception of CD86 even below the level expressed by monocytes without cytokine stimulation. Additionally, expression of CD14 was increased above monocyte level. All these data, together with the morphological observation, demonstrate that HAdV-infected monocytes cannot be differentiated into immature DC despite stimulation by IL-4 and GM-CSF.

DC are crucial for the induction of an adaptive immune response to viral infection. One of the DC subtypes, the mdDC directly differentiates from monocytes in the appropriate cytokine milieu. By the infection of this progenitor cell, HAdV is able to block differentiation to immature DC, thereby probably blocking the presentation of HAdV-derived antigens by these cells. Together with the potential to infect mature DC followed by a decreased expression of immunological activating surface markers, this could overcome the inability to impair expression of MHC and costimulatory molecules in HAdV-infected immature DC.

The ability to induce T-cell activation and proliferation is a hallmark of DC function. This ability summarizes all singular features like expression of MHC and costimulatory molecules, acquiring, processing and displaying antigens and secreting cytokines. Therefore, the success of viral subversion of DC function has to be measured by the virus' capability to evade T-cell proliferation. In order to assess the effect of adenoviral infection of mdDC on their T-cell stimulatory capacity, we performed a mixed lymphocyte reaction. DC were irradiated and co-cultured with autologous PBMC. After restimulation with inactivated viral particles, *in vitro* proliferation was detected with carboxyfluorescein succinimidyl ester (CFSE) (Feuchtinger *et al.*, 2008). Mock-infected DC stimulated 0.57 % of the autologous CD8^+^ T cells to proliferate, while direct stimulation of T cells with staphylococcus enterotoxin B resulted in a proliferation of 33 % of the CD8^+^ T cells. When HAdV-infected mdDC were incubated with the cells, 5.44 % of them proliferated (Fig. 3). This shows that the adenoviral infection of DC does not result in a block of CD8^+^ T-cell stimulation as can be seen in other viruses with similar impact on immune response. In comparison, HCMV leads to a remarkable decrease of the T-cell stimulatory capacity of infected DC (Grigoleit *et al.*, 2002; S. Schempp, personal communication). In contrast to this betaherpesvirus, HAdV-infected DC are still able to stimulate CD8^+^ T cells and thereby induce a cellular immune response.

## Figures and Tables

**Fig. 1.**
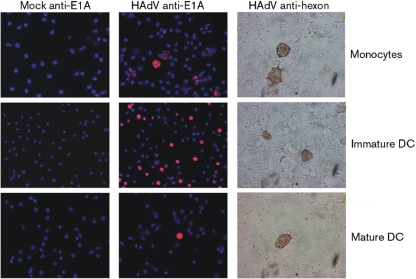
Expression of HAdV E and L antigens in infected cells (strain BB2000-61). Expression of E and L antigens was visualized 3 and 5 days post-infection. Cells were either mock infected or infected with BB2000-61. Mock is supernatant from cells treated exactly as infected ones except for the addition of HAdV. The m.o.i. of 1 resulted in 31 %, m.o.i. of 10 in 39 %, m.o.i. of 20 in 29 % and m.o.i. of 50 in 31 % positive-stained immature DC with E1A-staining. E1A antibody was diluted 1 : 100. Hexon specific antibody was diluted 1 : 200. Secondary antibody, anti-mouse immunoperoxidase was diluted 1 : 500. Counterstaining with DAPI.

**Fig. 2.**
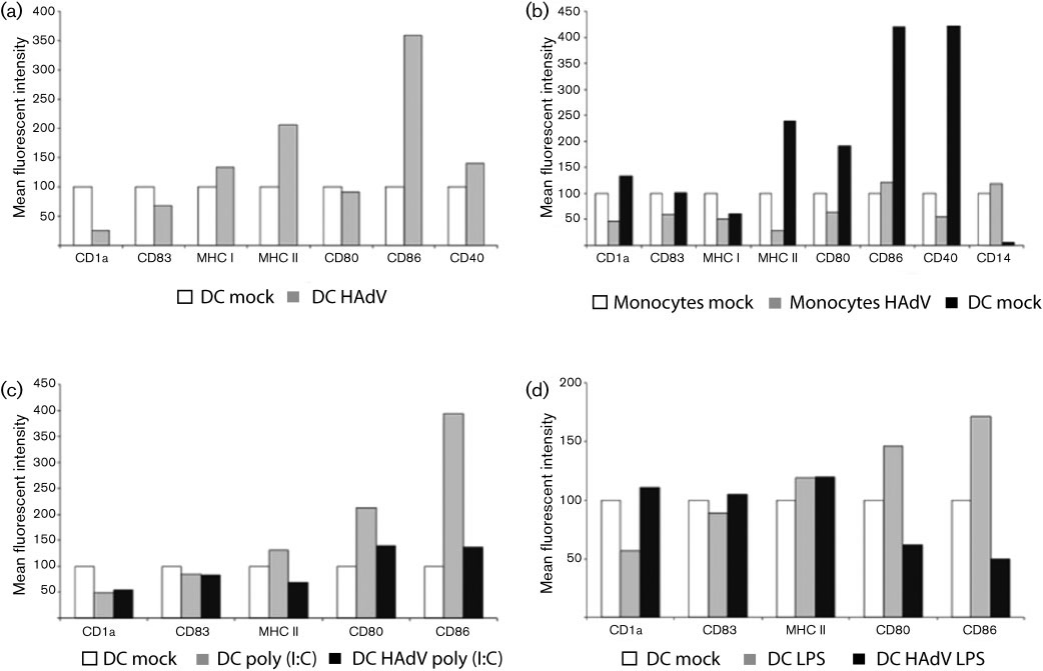
Expression of immunologically relevant surface markers after HAdV infection with an m.o.i. of 10. (a) Freshly isolated DC were stained with antibodies specific for the designated markers or differentiated into mdDC by the addition of IL-4 and GM-CSF and cultivation for 7 days, or infected with HAdV and cultivated for a further 7 days in the presence of IL-4 and GM-CSF then stained and analysed by flow cytometry. (b) Freshly isolated monocytes were infected with the strain BB2000-61 and cultivated for a further 5 days. (c, d) Mature DC were infected with the same virus. The experiments were performed twice.

**Fig. 3.**
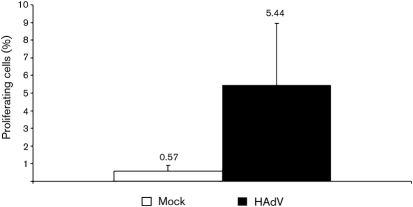
Stimulation of autologous T cells detected by CFSE. HAdV-infected DC were irradiated and co-cultured with autologous PBMC. Mock-infected DC stimulated 0.57 % of the autologous cytotoxic T cells to proliferate, while HAdV-infected mdDC induced an increase in proliferation to 5.44 %. Analysis of proliferation among cytotoxic T cells was done using a gate on CD3^+^/CD8^+^ cells.
